# Correlation between Traditional Chinese Medicine Constitution and Dyslipidemia: A Systematic Review and Meta-Analysis

**DOI:** 10.1155/2017/1896746

**Published:** 2017-10-22

**Authors:** Ye-lin Ma, Hui Yao, Wei-jia Yang, Xuan-xuan Ren, Long Teng, Min-chun Yang

**Affiliations:** Zhejiang Hospital, Hangzhou, Zhejiang 310013, China

## Abstract

**Objective:**

To study the correlation between Traditional Chinese Medicine (TCM) constitution and dyslipidemia.

**Methods:**

CNKI, VIP, Wanfang database, CBMdisc, PubMed, and Embase were searched, and meta-analysis was performed by Review Manager 5.2 software.

**Results:**

Altogether 11 studies were included with 12890 individuals. The results showed that balanced constitution was a protective factor of dyslipidemia (OR = 0.62, 95% CI 0.47~0.82) while phlegm-dampness constitution was a risk factor of it (OR = 2.50, 95% CI 2.22~2.80), and the effect of phlegm-dampness constitution in South China (OR = 3.31, 95% CI 1.71~6.43) was more obvious than that in East (OR = 2.40, 95% CI 2.06~2.80) and North China (OR = 2.24, 95% CI 1.81~2.78).

**Conclusion:**

This study provides evidence for the prevention and treatment of dyslipidemia in TCM. However, most of the studies included are of moderate quality; more high quality, multicenter, large-sample studies are expected to provide higher level evidence.

## 1. Introduction

Dyslipidemia refers to the abnormal quantity and quality of lipids in plasma, most of which result from the interaction between genetic defects and environmental factors. With the improvement of living standard and the change of life style, the prevalence rate of dyslipidemia in China has been significantly increased. According to “Chinese residents nutrition and health status (2004),” the prevalence of dyslipidemia in our country was 18.6%, estimated 160 million people. As a component of the metabolic syndrome, dyslipidemia can be accompanied with a variety of diseases, such as obesity, hypertension, hyperglycaemia, hyperuricemia, hyperinsulinemia, and fatty liver [1–3]. It is confirmed that high levels of cholesterol, chylomicron, low-density lipoprotein, and apolipoprotein B are risk factors of cardiovascular and cerebrovascular diseases such as atherosclerosis, coronary heart disease and ischemic stroke [4–7], and chronic kidney disease [8], which have negative effects on human health. The primary choice of therapy for controlling lipidemia has been statins, which are not completely effective [9, 10]. Screening for dyslipidemia in childhood could delay or reduce cardiovascular events in adulthood [11]. The theory of TCM has unique advantages in “preventive treatment of disease.” In recent years, a series of clinical studies based on the correlation between dyslipidemia and TCM constitution have been reported, which provide important clinical evidence for exploring the distribution of TCM constitution in dyslipidemia population, but there has been no systematic review or summary of the existing literature. This study carried out a meta-analysis of above studies, in order to obtain data based on larger samples and provide higher level evidence for clinical and scientific research.

## 2. Materials and Methods

This current meta-analysis was conducted following Meta-Analysis of Observational Studies in Epidemiology (MOOSE) [12] and Preferred Reporting Items for Systematic Reviews and Meta-Analysis (PRISMA) guidelines [13].

### 2.1. Search Strategy

Literature search was conducted by searching China national knowledge Internet database (CNKI), Chongqing VIP Chinese scientific journal database (VIP), Wanfang database, China Biology Medicine disc (CBMdisc), PubMed, and Embase, from the database creation to March 31, 2016. In Chinese database, [“dyslipidemia” or “hyperlipidemia”] + “constitution” were searched in title or abstract, and “Chinese medicine” was searched in full text or not limited field. In foreign database, search strategies were as follows: #1: dyslipidemia OR hyperlipemia; #2: constitution; #3: TCM OR Chinese medicine; #4: #1 AND #2 AND #3.

### 2.2. Inclusion/Exclusion Criteria

Inclusion criteria are as follows: ① research type: all clinical studies of correlation between TCM constitution and dyslipidemia (cross-sectional, case-control and cohort study, etc.), any nationality, written in Chinese or English; ② disease diagnosis: the research objects were definitely diagnosed as dyslipidemia; ③ constitution identification: constitution was identified by “Classification and determination of TCM constitution” [14] criteria published by China Association of Chinese Medicine (CACM) in 2009; ④ research results: the distribution of TCM constitution in dyslipidemia and ortholiposis population were both reported.

Literature with one of the following situations was excluded: ① the basic information of research object was not reported (gender, age and region, etc.); ② the research object suffered from acute coronary syndrome, cerebrovascular accident, cancer, or other life-threatening severe disease; ③ the research object suffered from serious mental disorder and failed to cooperate with researcher; ④ the research object had definitely compatible TCM constitution; ⑤ the results data were incomplete; ⑥ republished literature according to research data of the same population.

### 2.3. Literature Screening and Data Extraction

Literature screening and data extraction were conducted by 2 investigators independently, and the results of study were checked reciprocally. Study with divergence was discussed or determined by the 3rd investigator whether it was to be included or not. The data extracted mainly included researcher's name, time of publication, type of study, time and area of study, source of research object, sample size, results of study, and quality control measures.

### 2.4. Quality Evaluation

Cross-sectional study was evaluated by standard recommended by the United States Agency for Healthcare Research (AHRQ) [15], which was divided into 11 items with full mark 11 points, including data source, inclusion criteria, observation time, research object continuity, and quality control. Out of 11 points, 0–3 was divided into low quality, 4–7 medium quality, and 8–11 high quality. Case-control study and cohort study were evaluated by the Newcastle-Ottawa scale (NOS) [16], which was divided into 11 items of 3 aspects, including population selection, comparability between groups, and measurement of exposure factors. Out of 9 points, more than 6 points were divided into high quality.

### 2.5. Statistical Analysis

Meta-analysis was performed by Review Manager 5.2 software provided by Cochrane collaboration. Heterogeneity was tested by *I*^2^. If *I*^2^ ≥ 50% or *P* ≤ 0.05, there was heterogeneity and random-effects model was used. If *I*^2^ < 50% or *P* > 0.05, there was no heterogeneity and fixed-effect model was used. Effect size of each study was described by odds ratio (OR) and its 95% confidence interval (95% CI), and potential publication bias was tested by funnel plot. *P* < 0.05 was considered statistically significant.

## 3. Results

### 3.1. Literature Search

Altogether 112 articles were searched out from the databases initially, and 11 articles [17–27] were included finally according to inclusion and exclusion criteria, all of which were Chinese articles, 10 [17–24, 26, 27] of which were across-sectional studies and 1 [25] was a case-control study. A total of 12890 objects were included in the study, with 3909 cases of dyslipidemia (experimental group) and 8981 cases of ortholiposis (control group). The basic characteristics of the studies are listed in Table 1.

### 3.2. Meta-Analysis of Distribution of TCM Constitution in Dyslipidemia and Ortholiposis

#### 3.2.1. Balanced Constitution

**Figure 1:**
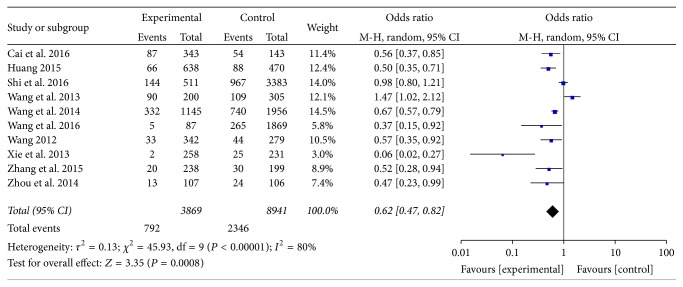
Meta-analysis of distribution of balanced constitution in dyslipidemia and ortholiposis.

Distribution of balanced constitution in dyslipidemia and ortholiposis people was reported in 10 articles [17–24, 26, 27]. There was heterogeneity among the studies (*I*^2^ = 80%, *P* < 0.05) and random-effects model was used. Meta-analysis showed that OR = 0.62, 95% CI [0.47, 0.82], and the difference was statistically significant (*P* = 0.0008). Refer to Figure 1.

#### 3.2.2. Phlegm-Dampness Constitution

**Figure 2:**
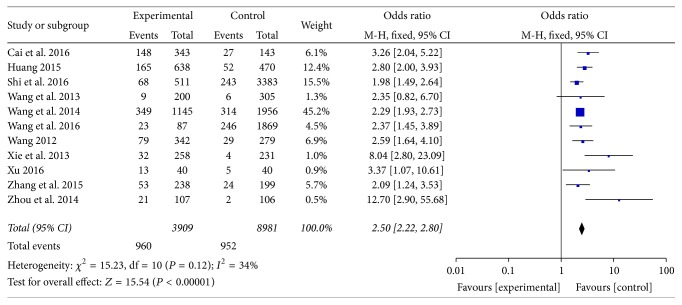
Meta-analysis of distribution of phlegm-dampness constitution in dyslipidemia and ortholiposis.

Distribution of phlegm-dampness constitution in dyslipidemia and ortholiposis people was reported in 11 articles [17–27]. There was no heterogeneity among the studies (*I*^2^ = 34%, *P* > 0.05) and fixed-effect model was used. Meta-analysis showed that OR = 2.50, 95% CI [2.22, 2.80], and the difference was statistically significant (*P* < 0.00001). Refer to Figure 2.

#### 3.2.3. Other TCM Constitutions

Distribution of qi deficiency constitution in dyslipidemia and ortholiposis people was reported in 10 articles [17–24, 26, 27], and yang deficiency, yin deficiency, damp-heat, qi stagnation, blood stasis, and allergic constitution were reported in 9 articles [18–24, 26, 27]. Meta-analysis showed that the difference was not statistically significant (*P* > 0.05). Refer to Table 2.

### 3.3. Meta-Analysis of Distribution of Phlegm-Dampness Constitution in Dyslipidemia and Ortholiposis in Different Areas

#### 3.3.1. North China

**Figure 3:**
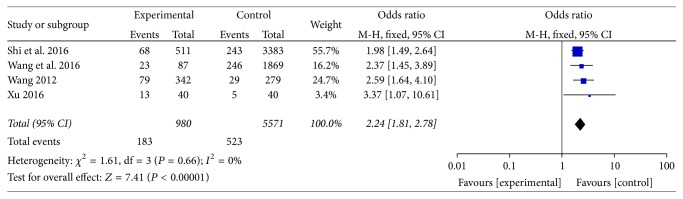
Meta-analysis of distribution of phlegm-dampness constitution in dyslipidemia and ortholiposis in North China.

Distribution of phlegm-dampness constitution in dyslipidemia and ortholiposis in North China were reported in 4 articles [19, 22, 23, 25]. There was no heterogeneity among the studies (*I*^2^ = 0%, *P* > 0.05) and fixed-effect model was used. Meta-analysis showed that OR = 2.24, 95% CI [1.81, 2.78], and the difference was statistically significant (*P* < 0.00001). Refer to Figure 3.

#### 3.3.2. East China

**Figure 4:**
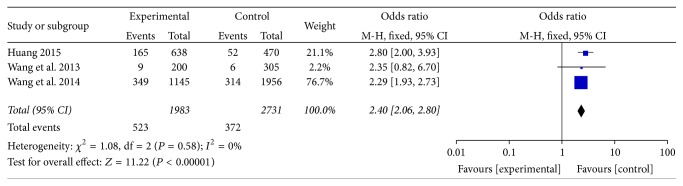
Meta-analysis of distribution of phlegm-dampness constitution in dyslipidemia and ortholiposis in East China.

Distribution of phlegm-dampness constitution in dyslipidemia and ortholiposis in East China were reported in 3 articles [18, 20, 21]. There was no heterogeneity among the studies (*I*^2^ = 0%, *P* > 0.05) and fixed-effect model was used. Meta-analysis showed that OR = 2.40, 95% CI [2.06, 2.80], and the difference was statistically significant (*P* < 0.00001). Refer to Figure 4.

#### 3.3.3. South China

**Figure 5:**
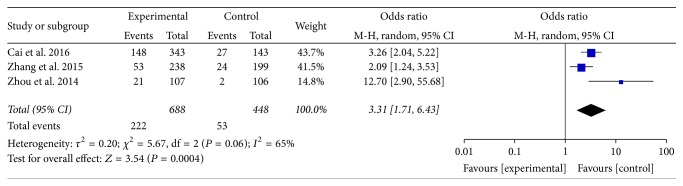
Meta-analysis of distribution of phlegm-dampness constitution in dyslipidemia and ortholiposis in South China.

Distribution of phlegm-dampness constitution in dyslipidemia and ortholiposis in South China were reported in 3 articles [17, 26, 27]. There was heterogeneity among the studies (*I*^2^ = 65%, *P* > 0.05) and random-effects model was used. Meta-analysis showed that OR = 3.31, 95% CI [1.71, 6.43], and the difference was statistically significant (*P* = 0.0004). Refer to Figure 5.

### 3.4. Analysis of Publication Bias

**Figure 6:**
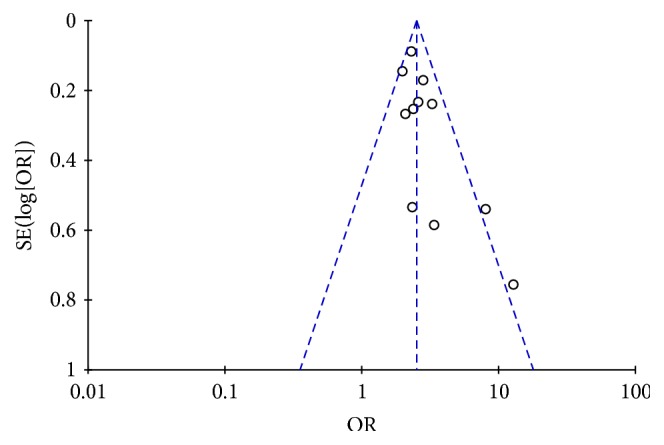
Funnel Plot of publication bias of studies included.

Funnel plot of the 11 studies showed that the symmetry was acceptable, and most of the splashes were in the funnel, which indicated that the publication bias had little effect on the results of meta-analysis. Refer to Figure 6.

## 4. Discussion

“Dyslipidemia” can be classified as “phlegm,” “wet,” or “blood turbidity” in Traditional Chinese Medicine, which is caused by overeating greasy food, dysfunction of transportation, and transformation of spleen and stomach, and stagnation of qi and phlegm-dampness. It is believed that deficiency of spleen and kidney is the basis of “hyperlipemia,” and phlegm-dampness and blood stasis are the representation of it [28]. The dampness evil enters into the blood and circulates through the body, which is similar to the rise of TG, TC, and LDL-C in blood in western medicine. Guo et al. [29] searched modern literature and found that herbs such as* Rheum officinale* (Dahuang), Rhizoma Polygonum cuspidate (Huzhang), Semen Cassia (Juemingzi),* Coptis chinensis* (Huanglian),* Scutellaria baicalensis* (Huangqin),* Gynostemma pentaphyllum* (Jiaogulan), Radix Puerariae (Gegen), Fructus crataegi (Shanzha), and Red yeast rice (Hongqu) were frequently used in treatment of hyperlipemia and achieved significant effect in reducing TG, TC, and LDL-C in blood. Chen et al. [30] treated hyperlipemia with self-made prescription (hawthorn, Salvia, rhizoma alismatis, Polygonum multiflorum, cassia seed, etc.) combined with simvastatin and achieved significant effect in reducing blood fat and improving hemorheology and coagulation function.

The TCM constitution refers to the comprehensive, relatively stable, and inherent characteristics of the morphological structure, physiological function, and psychological state formed on the basis of innate endowment and acquired disposition in the course of human life [14]. It is a human personality characteristic formed in the process of human growth and development, which adapts to the natural and social environment. According to “Classification and determination of TCM constitution” published by China Association of Chinese Medicine (CACM) in 2009, the TCM constitution can be classified into nine basic types: balanced, qi deficiency, yang deficiency, yin deficiency, phlegm-dampness, damp-heat, blood stasis, qi stagnation, and allergic constitution. Among them, the balanced constitution is a normal constitution, while the other eight constitutions are biased constitutions. Different types of constitutions have their own characteristics in physical characteristics, physiological characteristics, psychological characteristics, pathological reaction states, morbidity tendency, and so on.

For example, the balanced constitution, formed due to the harmony of yin, yang, qi, and blood, is characterized by moderate posture, ruddy complexion, and vigorous energy. People with balanced constitution are generally easygoing and cheerful and do not suffer easily from disease, with strong adaptability to natural and social environment, while the phlegm-dampness constitution, formed due to the accumulation of phlegm and dampness, is characterized by fat posture, flabby abdomen, sticky mouth, and greasy coating on the tongue. People with phlegm-dampness constitution are generally gentle, steady, and patient and easily suffer from disease such as diabetes mellitus, apoplexy, and coronary heart disease, with weak adaptability to rainy season and moist environment.

The results of meta-analysis showed that balanced constitution was a protective factor of dyslipidemia (OR = 0.62, 95% CI 0.47~0.82), which conformed to the theory “only when yin is mind and yang is compact can essence and spirit be normal” in “Plain Questions,” while phlegm-dampness constitution was a risk factor of it (OR = 2.50, 95% CI 2.22~2.80), which generally conformed to above discussion of etiology and pathogenesis of “blood turbidity” in TCM. The results of subgroup meta-analysis according to the region showed that OR value was in successively South China (OR = 3.31, 95% CI 1.71~6.43), East China (OR = 2.40, 95% CI 2.06~2.80), and North China (OR = 2.24, 95% CI 1.81~2.78) from high to low, which might be related to the climate characteristics. The climate in South China is warm and humid, which may easily generate phlegm, dampness, and heat inside the bodies of local residents; then the real evils are mixed into the blood and transformed into “blood turbid,” while the climate in North China is relatively cool and dry, which may not easily generate phlegm or dampness, thus having less influence on health of local residents. The results also showed that blood stasis (OR = 1.48, 95% CI 0.80~2.74), qi deficiency (OR = 1.10, 95% CI 0.87~1.39), and damp-heat constitution (OR = 1.06, 95% CI 0.76~1.49) seemed to have some positive correlation with dyslipidemia, but the difference did not show statistical significance (*P* > 0.05). More large-sample studies are still needed for verification.

Most of the 11 studies included were of moderate quality according to quality evaluation (Referring to Table 1). Taking 10 cross-sectional studies [17–24, 26, 27], for example, definite data sources and inclusion/exclusion criteria of experimental/control group were reported in 10 studies [17–24, 26, 27], the time phasing for patients identification was reported in 9 studies [17–19, 21–24, 26, 27], the measures for confounding factors evaluation and control were reported in 8 studies [17–23, 26], and the response rate and completeness of data collection were reported in only 1 study [23], while the continuity of objects, subjective factors of researchers, and processing of missing data were not reported in any studies. The cross-sectional studies included had significant heterogeneity, so there might be some risk of bias in the results. But it is considered that there are differences in TCM constitution caused by region, gender, and age factors according to TCM theory [31], which is different from the heterogeneity of clinical trials. Therefore, in order to describe distribution characteristics of TCM constitution in dyslipidemia people of the whole country, this study still included the original literature data into the meta-analysis and further carried out subgroup meta-analysis according to the region, in order to obtain more accurate results.

In summary, this study carried out meta-analysis of related literatures and obtained the data of distribution of TCM constitution in dyslipidemia population based on 12890 cases of large sample, which provided certain evidence for the clinical and scientific research. But there are still some limitations: (1) most of the studies included are cross-sectional study, and the quality remains to be further improved, in order to reduce the risk of bias caused by human factors. (2) Considering the heterogeneity of TCM constitution itself, subgroup research according to region, gender and age, and so on should be conducted except for description of the distribution characteristics of TCM constitution of the whole sample. More high quality, multicenter, and large-sample studies are expected to provide higher level evidence for the clinical and scientific research.

## Figures and Tables

**Table 1:** The basic characteristics and quality score of studies included.

Study ID	Type	Area	Source	Period	Sample size (experimental/control)	Constitution	Quality score
Cai et al. 2016 [17]	CS	Lingnan	PE	2014.6–2015.2	486 (343/143)	Balanced, Qi-deficiency, phlegm-dampness	4
Huang 2015 [18]	CS	Shanghai	PE	2013-2014	1108 (638/470)	9 types	4
Shi et al. 2016 [19]	CS	Beijing	PE	2014.1–2014.12	3894 (511/3383)	9 types	4
Wang et al. 2013 [20]	CS	Anhui	PE	Unknown	505 (200/305)	9 types	3
Wang et al. 2014 [21]	CS	Ningbo	PE	2012.10–2013.5	3101 (1145/1956)	9 types	4
Wang et al. 2016 [22]	CS	Beijing	OP	2013.11–2014.9	1956 (87/1869)	9 types	4
Wang 2012 [23]	CS	Beijing	PE&OP	2008–2011	621 (342/279)	9 types	5
Xie et al. 2013 [24]	CS	Wuhan	OP&IP	2009-2010	489 (258/231)	9 types	3
Xu 2016 [25]	CC	Beijing	PE	2014.12–2015.12	80 (40/40)	Phlegm-dampness	5
Zhang et al. 2015 [26]	CS	Guangzhou	PE	2012	437 (238/199)	9 types	4
Zhou et al. 2014 [27]	CS	Guangzhou	PE	2013.1–2014.1	213 (107/106)	9 types	3

*Note*. CS: cross-sectional study; CC: case-control study; PE: physical examination; OP: outpatient; IP: Inpatient.

**Table 2:** Meta-analysis of distribution of other TCM constitutions in dyslipidemia and ortholiposis.

TCM constitution	Sample size (case)		Heterogeneity test		OR	95% CI	*Z*	*P*
	Experimental	Control	*I* ^2^ (%)	*P*				
Qi deficiency	3869	8941	71	0.0003	1.10	0.87~1.39	0.78	0.44
Yang deficiency	3526	8798	69	0.001	0.83	0.62~1.10	1.29	0.20
Yin deficiency	3526	8798	73	0.0003	0.74	0.53~1.02	1.85	0.06
Damp-heat	3526	8798	67	0.002	1.06	0.76~1.49	0.35	0.72
Qi stagnation	3526	8798	69	0.001	0.76	0.53~1.10	1.46	0.14
Blood stasis	3526	8798	91	<0.00001	1.48	0.80~2.74	1.23	0.22
Allergic	3526	8798	53	0.03	0.90	0.61~1.31	0.57	0.57
